# Interpersonal psychotherapy for traumatic grief following a loss due to COVID-19: a case report

**DOI:** 10.3389/fpsyt.2023.1218715

**Published:** 2023-09-29

**Authors:** Farah Deena Abdul Samad, Xavier Vincent Pereira, Siew Koon Chong, Muhammad Hanif Bin Abdul Latif

**Affiliations:** ^1^Department of Psychiatry, Faculty of Medicine, National University of Malaysia, Kuala Lumpur, Malaysia; ^2^Taylor’s University School of Medicine, Malaysia and Health Equity Initiatives, Subang Jaya, Malaysia; ^3^Department of Psychiatry, Hospital Sultanah Nur Zahirah, Kuala Terengganu, Malaysia

**Keywords:** traumatic grief, interpersonal psychotherapy, posttraumatic stress disorder, COVID-19 pandemic, grief and loss, complicated grief

## Abstract

Interpersonal psychotherapy (IPT) is a highly regarded evidence-based psychotherapy that aims to alleviate the suffering of clients and improve their interpersonal functioning. Research has demonstrated the effectiveness of IPT in depressive, bipolar and eating disorders. IPT also focuses on grief and loss as a problem area to help clients address and process their grief symptoms, leading them to reach a phase of finding meaning. However, traumatic grief which is characterized by someone who has both symptoms of trauma and grief can further complicate treatment. As for Posttraumatic Stress Disorder (PTSD), IPT can be a choice of treatment by addressing perceived isolation and emotional dysregulation through mobilizing adequate social support. This case study highlights the efficacy of IPT in treating complicated grief with traumatic experiences caused by the loss of a loved one during the COVID-19 pandemic, without undergoing exposure-based therapy. The treatment course consisted of 12 sessions scheduled twice weekly, and the client received antidepressant medication augmented with antipsychotic medication. After undergoing IPT, the client experienced an improvement in symptoms, gradual recovery of functional disability, and more meaningful interpersonal relationships. The case study presented provides evidence to suggest that IPT is a promising treatment approach for individuals struggling with trauma related to grief.

## Introduction

Interpersonal therapy (IPT) is a psychological therapy that was created by Gerald Klerman and Myrna Weissman, which focuses on clients’ social and interpersonal functioning, emotion, and ongoing life events (1). IPT can help the client understand their difficulties through social and interpersonal aspects that were based on the psychosocial theory by Harry Stack Sullivan and John Bowlby. Prior research has demonstrated that IPT can be effective for individuals with bipolar disorder, depression, and eating disorders (2, 3). As one of the important parts of IPT, grief can also be targeted during the therapy to help the client process the condition and reach the phase of finding meaning, as explained by David Kessler (4). However, if integration of grief process failed, it will lead to Persistent Complex Bereavement Disorder or complicated grief. Those with complicated grief, often has co-morbidity with Major Depressive Disorder (MDD) and Posttraumatic Stress Disorder (PTSD) which further complicates treatment (5). A systematic review and meta-analysis showed that clients with Posttraumatic Stress Disorder (PTSD) can also benefit from IPT sessions, although not as a first line of treatment (2, 6). IPT can be the choice of treatment for clients who are not responding or cannot tolerate exposure-based therapy, which could exacerbate the symptoms (7). The role of IPT in treatment of complicated grief is useful in patients with attachment rupture and inadequate social support (2). Among the main symptoms are impaired interpersonal functioning, which could lead to perceived isolation and emotional dysregulation. These two symptoms can be addressed by the elements of IPT, which focus on improving the relationship with others that eventually provide adequate social support to the clients. During the pandemic COVID-19, many people lost their loved ones to illness worldwide. The losses happened in unusual circumstances that happened very quickly and unexpectedly. Most of them did not go through the grieving process properly, which led to complicated grief (8). Therefore, we present a case of complicated grief with traumatic experiences who lost her loved one during the COVID-19 pandemic and received antidepressant and IPT treatment for her condition without undergoing exposure-based therapy to reduce the symptoms.

## Case description

### Case presentation

Miss S, a 37-year-old Malay female, worked as a lecturer and presented to our clinic with depression and traumatic symptoms. These symptoms started a year ago when she lost her fiancé, due to COVID-19. It was an unexpected event that happened all too quickly.

Miss S recalled undergoing quarantine with her fiancé as they had both tested positive for COVID-19. On the sixth day of the quarantine, her fiancé’s condition deteriorated to high-grade fever and worsening cough. She decided to bring him to medical attention after seeing him gasping for breath. Miss S was unable to hail an ambulance as most of them were already in demand. In desperation, she rushed him to the hospital herself after seeing critical oxygenation levels on a self-monitoring device. Miss S described the harrowing event with great distress and difficulty. She described the panic-like sensation of seeing her boyfriend deteriorate before her very eyes; being breathless, gasping for air, and then becoming unresponsive. He was pronounced dead upon arrival at the emergency department. For Miss S, the ordeal was far from over. After the incident, she developed recurrent intrusive and distressing memories about the death. She was troubled by the constant thought of what she could have done better to prevent the incident and suffered from a dreaded sense of guilt. She also began suffering from nightmares and flashbacks of the incident; of the morbid and pale features of her fiancé’s final moments before his demise at the back of her car. Even the sight of his belongings at home was sufficient to trigger unpleasant palpitations.

Miss S began exhibiting salient lifestyle changes ever since. She actively avoided using the roads heading to the hospital and refused to listen to any news relating to morbidity. At work, her colleagues noticed that she became more edgy and was easily startled by subtle stressors, some causing her to develop panic-like symptoms with shortness of breath and palpitations. She was constantly spacing out and her concentration at work was poor. As her performance declined, Miss S gradually withdrew into a shell and refused to socialize altogether. She described a pervasive sad mood with no interest in most activities. She was no longer her usual joyful self and had no appetite to eat. As for Miss S, she described an impending sense of dread that keeps her on her toes most of the time. This sense of hypervigilance worsens during nightfall and Miss S developed a particular fear of the dark. She became insecure and started video-calling her parents who lived overseas.

Sensing that something was amiss, her parents decided to visit her. They too, noted that Miss S was a changed person. She spoke constantly of her feelings of guilt and wished that she was granted death to be reunited with her fiancé in the afterlife. Concerned with her intense suicidal thoughts, Miss S was eventually brought to psychiatric attention.

### Diagnostic assessment

Miss S had been diagnosed with Persistent Complex Bereavement Disorder (complicated grief) with traumatic bereavement, with co-morbid Major Depressive Disorder. Upon assessment, Miss S expressed suicidal thoughts as she felt hopeless of the future and wish to join her fiancé in the afterlife. She had no plans, no access to means and no history of substance usage. Her protective factor was her religion and parents. She had no past medical and psychiatric illness. There we no substance usage. She had done a psychological tools assessment using PTSD Checklist (PCL5) and Patient Health Questionnaire (PHQ). For PCL5, she scored 15/20 for re-experiencing, 8/8 for avoidance, 20/28 for negative alteration in cognition and mood, and 16/24 for hyper-arousal. Meanwhile, for PHQ-9, her score was 19, which indicated moderate to severe severity. Physical examination was unremarkable.

### Treatment course

The patient received 12 IPT sessions, each lasting 60 min. Sessions were conducted once weekly or once every 2 weeks. The first author (FD) delivered the treatment, having been trained by and in the supervision of XP, an International Society of Interpersonal Psychotherapy (ISIPT) certified trainer/supervisor. The patient was started on Sertraline 150 mg daily for her depressive symptoms which was subsequently augmented with Aripiprazole 5 mg daily on the 11th session.

### Initial phase (sessions 1 to 3)

During the first session of interpersonal psychotherapy, the therapist reviewed the client’s complex grief and PTSD symptoms, assessing the extent of her functional impairment. The client tearfully shared that she struggled with flashbacks, a mixed sense of confusion yet feeling extremely guilty at the same time. Here, the therapist helped the client identify and link her feelings (sad, lonely, loss, guilt, scared) which is the first important step in identifying and resolving her trauma and grief. Miss S felt relieved of being able to talk freely about her late partner as she felt very lonely throughout the entire ordeal. In this session, a therapeutic contract was established.

**Figure 1:**
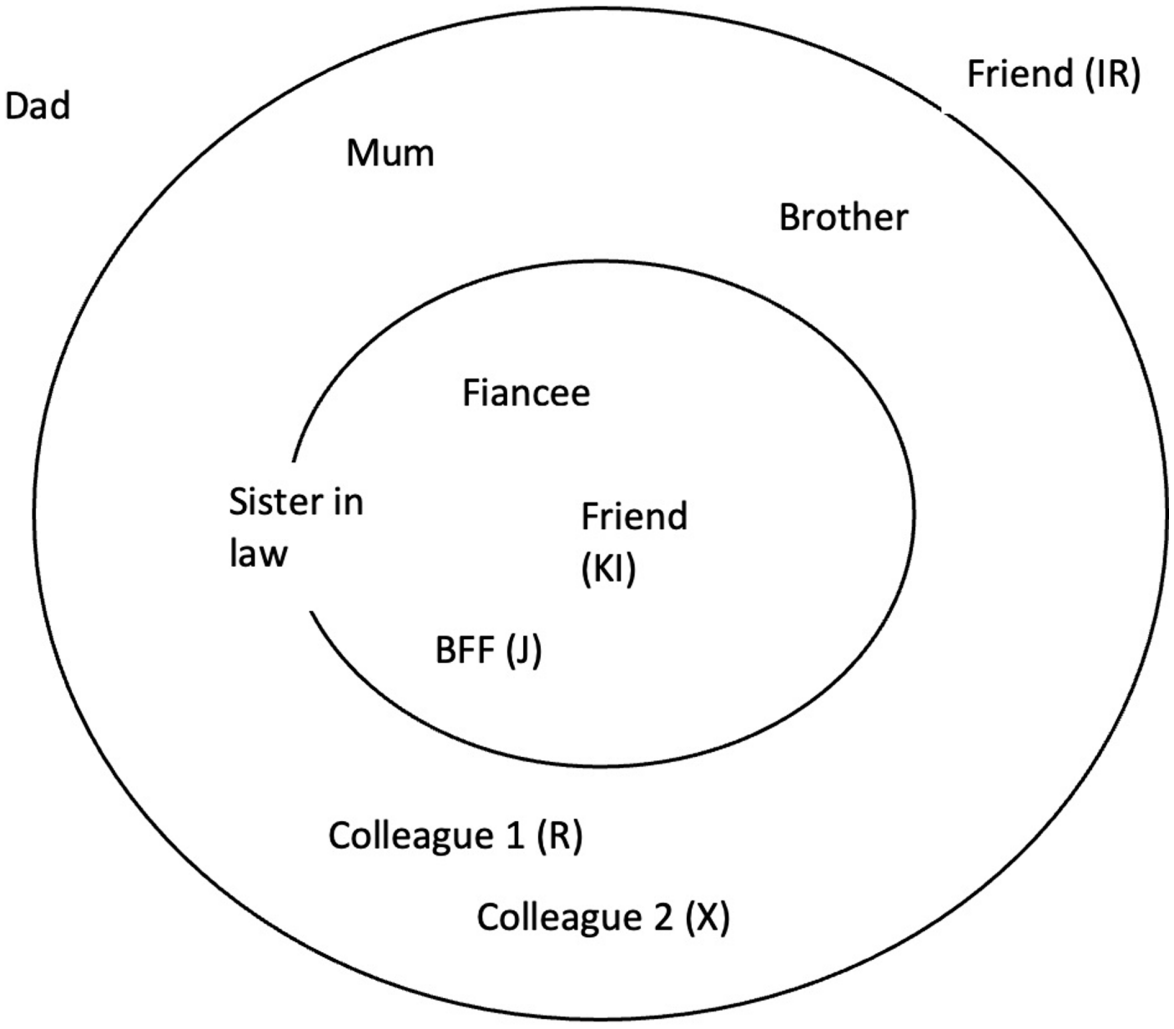
Interpersonal inventory.

The second and third sessions focused on conducting an interpersonal inventory (Figure 1) and case formulation. The client began by mapping out important relationships in her life, identifying her late fiancé, best friend, ex-colleague, and fiancé’s sister being the most important.

Both the positive and negative aspects of these relationships were explored. The client painted a positive picture of her late fiancé. He was depicted as calm, laid-back, and financially dependable. Miss S had plans of getting married to him and build a family. With the passing of her fiancé, these plans and hopes are now lost. Miss S was also disappointed with the reaction of her friends who attributed her condition to the lack of religious affinity. When asked why Miss S chose to place her parents as distant social contacts, Miss S revealed that she was frustrated that they lived far away and were unable to provide adequate support.

**Figure 2:**
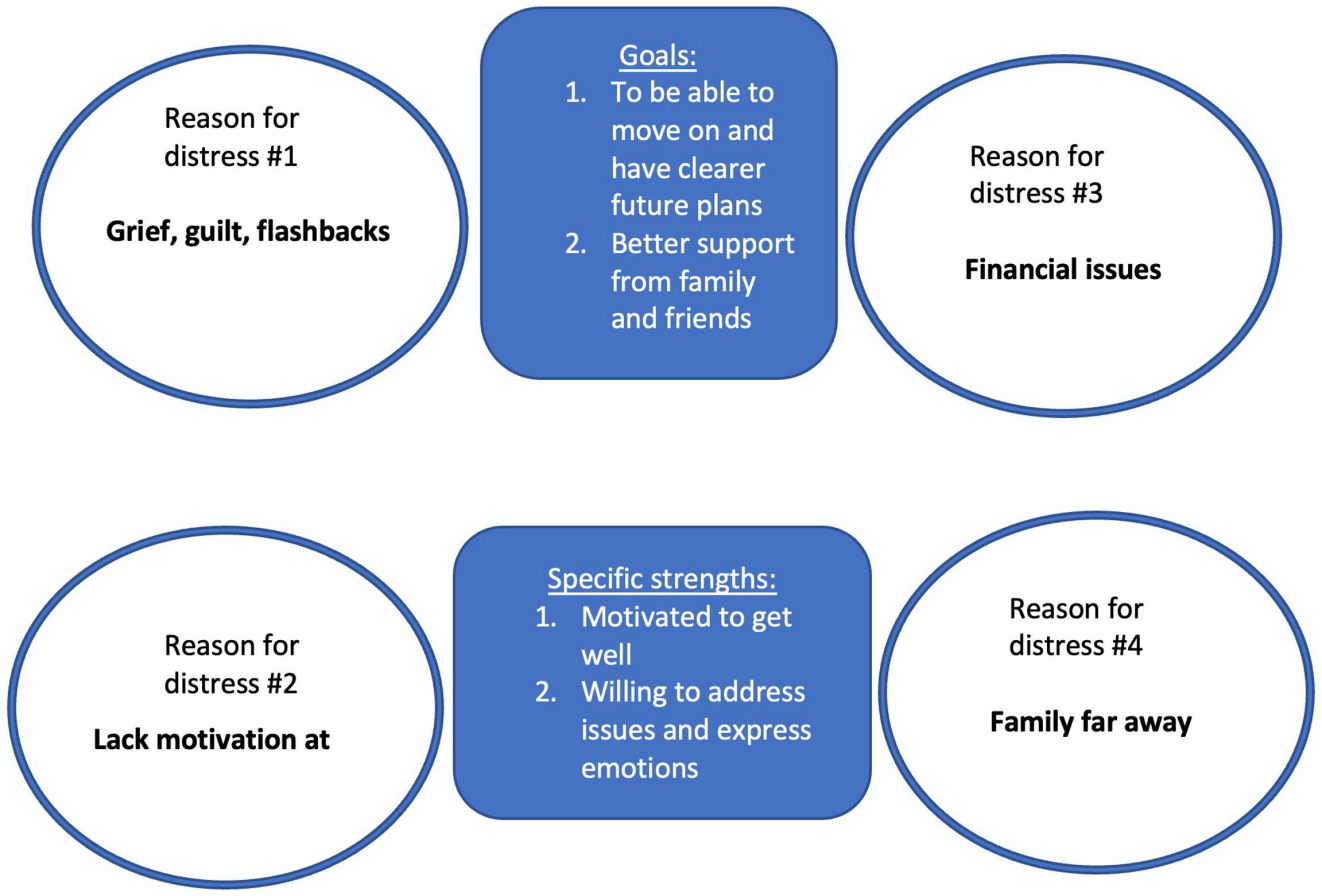
Interpersonal formulation.

The therapist summarized that her fiancée was indeed a significant figure who provided her with emotional stability and acted as a grounding force. It was understandable that his loss would cause a substantial void, one which Miss S was struggling to accommodate. On a positive note, Miss S still possessed good motivation to recover. Being able to access and identify difficult emotions, good insight, and her willingness to follow through with therapy point toward a good prognosis for this client (Figure 2).

### Middle phase (sessions 4–9)

During our fourth session, the client reported feeling well-rested after a week of medical leave. Miss S spoke about the recent first anniversary of her fiancé’s death. Fortunately, she had been coping well. Miss S had a dream about her fiancée visiting her. The encounter left her feeling happy and comforted. She kept herself busy throughout the day by arranging a religious memorial ceremony online, visiting his grave, and reconnecting with old friends who had been an important part of her life with her fiancée. She was able to attend a pleasant dinner with a friend who was not in her interpersonal inventory. The therapist highlighted the positive mood shift and the client’s ability to reconnect with her social network as a good sign of progress.

The next challenge was to facilitate Miss S’s endeavor to seek closure for her grief. We began by recollecting and reflecting on happy memories with her fiancé. Miss S spoke fondly of the first time they met and detailed her fiancé’s charming demeanor.

Subsequent sessions gradually transitioned into the circumstances which led to her fiancé’s demise. Here, we postulate that facilitated reminiscence of the traumatic encounter would aid Miss S in finding some closure to her grief.

Miss S began by detailing her feelings of anxiety and fear as she rushed her fiancé to the hospital. It was then that Miss S gradually disclosed that she was initially reluctant to bring her fiancé to seek medical attention despite her fears that he was already deteriorating. She also blamed herself for not keeping an eye on the pulse oximeter and was troubled by the dreaded sense that her complacency was what led to his death.

We decided that some role play would be helpful to aid Miss S in constructively expressing her feelings. Here, the therapist assumes the role of the deceased, and Miss S would be allowed to share what she would have said to the late fiancé.

*The therapist: What would you say to him about the quarantine?*

*“I’m sorry I did not do a lot. I was sick too, but I wasn’t as sick as you. My brain was so slow at that time, I could not think clearly. I wished I had forced you to go to the hospital earlier. And I’m sorry I did not realize it was your last moments in the car, I was scared, I was driving, I was looking at Waze. I did not do as much as I could have. Part of the cause of your death is me, my brains were slow, I did not do a lot of things, and I could have fought it if I knew it was your last day, but I did not do a lot of things. It’s not your fault, you could not think at that time, you were unwell, I was supposed to decide for you.”*

Expressing her feelings of guilt aided Miss S greatly. Miss S admitted feeling a sense of relief as if a great burden had been lifted. We believe that Miss S suffered from a lot of pent-up frustration and distress as she isolated herself from society. This was compounded by the sudden passing of her fiancé without her being able to bid a proper farewell. Role play in this instance served as a medium of catharsis for our client, giving Miss S the solace and closure that she was seeking for.

Subsequent follow-up saw substantial improvement in Miss S. She was able to engage in more productive activities such as socializing. Miss S began contacting her parents and her best friend to seek support at times of need. If recollecting the stories of her previous trauma causes her significant distress, now Miss S was able to share her struggles with an online support group. External cues and reminders of her late fiancé still caused her some distress, albeit at a lesser intensity.

By the end of the middle phase, her PHQ-9 scores have reduced from 19 to 15 and PCL-5 from 59 to 30.

### Termination phase (sessions 10–12)

During therapy, the patient and therapist explored the patient’s relationships and social support. We identified her attachment as the anxious type. It is expected for Miss S to undergo an ordeal of distress in her attempt to shift to a new role, devoid of her fiancé. As Miss S is more stable now, the termination phase focuses on her mobilizing available social networks to form enduring support. She was able to verbalize her needs and reach out to her friends and family for help. For instance, Miss S felt more willing to engage her parents which is now viewed as a close contact at times of need. Miss S and her fiancé used to love food hunting, now she has her own set of friends whom she is able to reach out for her love of food. At times, they would revisit the places she used to go with her fiancé and she felt happy reminiscing the memories they had.

As for herself, Miss S uncovered a fresh array of hobbies and abilities, including cooking, a task that was formerly undertaken by her fiancé. She moved in with her best friend, who also shared a strong bond with her fiancé. Every Sunday, she continues visiting his graveyard and keep him updated regarding her life. The patient acknowledged that her traumatic symptoms no longer affected her daily functioning. However, she felt a lack of motivation and concentration persisted albeit at a lesser intensity than the initial presentation. After careful consideration, we decided to aid Miss S’s recovery by augmenting with Aripiprazole 5 mg as she could not tolerate increased dose of Sertraline.

By the conclusion of the therapy session, her PHQ-9 scores had decreased from 15 to 5, and her PCL-5 scores had gone down from 30 to 13. During the subsequent maintenance sessions, scheduled 2 months apart, Miss S maintained a positive outlook and expressed satisfaction with the progress she had achieved. She found that being able to openly share her feelings about her traumatic loss and reminisce about her fiancé with the therapist was therapeutic and played a significant role in her recovery journey. This experience allowed her to open up more easily and connect with others about her struggles, something she had struggled with in the past.

**Figure 3:**
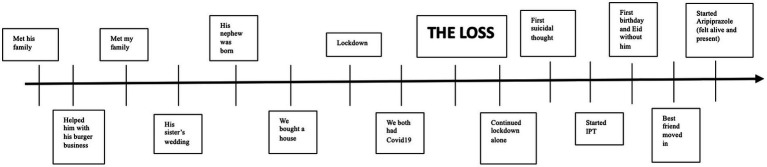
Life events timeline.

Participating in IPT highlighted for her the effective utilization of her social support network during challenging times. Moreover, gaining a deeper understanding of her anxious attachment style further enriched her self-awareness. Given her strong insight and favorable response to both medication and therapy, her prognosis appears promising (Figure 3).

## Discussion

Interpersonal Psychotherapy (IPT) was initially derived from the theoretical work of Harry Stack Sullivan and John Bowlby and empirical research on the psychosocial aspects of depression (1). Sullivan viewed interactions with others as the most profound source of understanding one’s emotions, while Bowlby considered relationships and affection with others as an important aspect to maintain psychological well-being (9). With these concepts in mind, IPT practitioners have begun to find new applications for IPT, especially when dealing with other psychiatric illnesses such as PTSD. Some case studies have also found the clinical utility of IPT in the treatment of grief (3).

In this report, we emphasize the distinctive role played by IPT in facilitating our client’s recovery through guided sessions. The first focus was on resolving her grief, while the second centered on helping her navigate the aftermath of the traumatic experience and adjust to a new life role.

### Trauma

Initial evidence suggests that IPT may also benefit patients with post-traumatic stress disorder PTSD (3). There are at least two rationales for utilizing IPT for patients with PTSD. Firstly, IPT does not utilize exposure to trauma reminders. Although more exposure-based therapies for PTSD have more empirical support (10) IPT offers an alternative to patients who prefer a more gradual approach. In this case, we find that Miss S, being a patient who suffers from flashbacks, and disturbing nightmares and actively avoids them, would probably benefit from a therapy that assumes a more gradual approach. This is achieved by focusing on the “here-and-now” problems rather than childhood or developmental issues (1, 7). Sessions open with the question “How have things been since we last met?” This focuses the patient on recent interpersonal events and recent mood, which the therapist attempts to link. Therapists take an active, non-neutral, supportive, and hopeful stance (2).

Recent evidence also suggested that highly traumatized patients may respond better to affect-focused as compared to more traditional exposure-based therapy (7) especially those who suffer from dissociation and traumatic flashback such as seen in the case of Miss S. Secondly, IPT works by improving patients’ interpersonal functioning and emotion regulation (2, 6), which are commonly impaired in traumatized patients and therefore, are important targets for change. Social support, which IPT helps patients to mobilize, is a key factor in preventing and recovering from trauma (11, 12) as compared to trauma-focused CBT which places specific emphasis on cognitive distortions.

### Grief and role transition

The present study examines three aspects of interpersonal functioning that serve as predictors of grief therapy outcomes. These are the patient’s (1) attachment to the lost person, (2) quality of object relations, and (3) level of recent social role functioning. Each variable provides a unique perspective on an individual’s interpersonal functioning and may affect the outcome of grief therapy through different mechanisms (1, 2).

One main theme surrounding grief which many patients struggle to overcome is processing and accepting loss, usually about a significant figure in the individual’s life. Complicated grief is significantly less in those with secure attachment (13). As seen in Miss S, she developed complicated grief likely contributed by her anxious attachment by causing heightened dependency on the deceased, intensifying fears of abandonment, triggering rumination and preoccupation, disrupting emotional regulation and limited effective coping mechanism. IPT shares many themes in common with grief therapy where one evaluates the bond with the loved one, the significance of the loss, and finding new meaning in the aftermath (4, 14). Complicated Grief Therapy (CGT), which integrated IPT and Cognitive Behavioral Therapy (CBT), demonstrated greater efficacy in addressing complicated grief compared to using IPT alone (15). Miss S’s situation was compounded by her social isolation following an 8-year relationship, exacerbated by the traumatic loss of her fiancé during a Movement Restricted Order due to the COVID-19 pandemic. Additionally, she faced significant panic attacks when confronted with reminders of her fiancé’s traumatic death, making cognitive reframing and exposure techniques less suitable at that specific time.

The influence of social role functioning on the outcome of grief therapy has not been previously examined; however, the works suggest that it may be important (4, 14). They argue that when a loved one dies, new social role responsibilities (e.g., child care, financial management) are passed on to the bereaved person. Some individuals are not able to fullfil these responsibilities (14).

In a role transition, a life change costs the patient an old role and substitutes for a new, unwanted one. Treatment helps the patient mourn the loss of the former and develop skills, interpersonal opportunities, and confidence in the latter, new role. The patient deals with the change by recognizing the positive and negative aspects of the new role he or she is assuming assets and liabilities of the old role this replaces (2). Interpersonal deficits, the residual fourth IPT problem area, define the patient as significantly lacking in social skills, including initiating or sustaining relationships with significant others (16).

### Role of antidepressants

Overcoming post-traumatic grief using IPT requires multiple sessions and a strong commitment from both therapist and client. Antidepressant such as Sertraline is used to hasten recovery from depression as well as emotional distress especially in the early phase of treatment (1, 2). Severely distraught clients may not be able to cooperate with IPT, especially those in the acute phase of the crisis. We believe that reduction of psychological distress would somehow enable clients to attend IPT sessions with greater mental clarity. In the case of Miss S, the use of Sertraline, augmented with a low dose of Aripiprazole (17) had aided greatly in facilitating her recovery.

## Conclusion

This report highlights how IPT effectively aided patients’ recovery from grief and post-traumatic stress disorder. Its clinical utility in helping a patient reconcile lost bonds, forging new ones, and adapting to new roles after a crisis appears to be very useful. Although accepting a loss and adapting is a painful and arduous process, we believe that most patients already possess the resources and social connections required to overcome a particular crisis. Unfortunately, this innate potential becomes obscure when powerful emotions evoked during a crisis take center stage. Hence our role as therapists is to help our patients realize, mobilize and utilize their available social resources to facilitate their recovery.

## Data availability statement

The raw data supporting the conclusions of this article will be made available by the authors, without undue reservation.

## Ethics statement

Ethical approval was not required for the studies involving humans because Ethics review and approval/written informed consent was not required as per local legislation and institutional requirements. The studies were conducted in accordance with the local legislation and institutional requirements. The participants provided their written informed consent to participate in this study. Written informed consent was obtained from the participant/patient(s) for the publication of this case report.

## Author contributions

FA conducted IPT on the client and contributed to the conceptualization and writing of the manuscript. XP supervised the IPT sessions and conceptualization of the manuscript. SC contributed to the conceptualization and writing of the manuscript. MA worked with the patient and her family and contributed to the writing. All authors have approved the submitted version.

## References

[1] Weissman MM. Interpersonal psychotherapy: history and future. Am J Psychother. (2020) 73:3–7. doi: 10.1176/appi.psychotherapy.20190032, PMID: 31752510

[2] Markowitz JC Weissman MM. Interpersonal psychotherapy: principles and applications. World Psychiatry. (2004) 3:136–9. PMID: 16633477 PMC1414693

[3] Rafaeli AK Markowitz JC. Interpersonal psychotherapy (IPT) for PTSD: a case study. Am J Psychother. (2011) 65:205–23. doi: 10.1176/appi.psychotherapy.2011.65.3.205, PMID: 22032045 PMC3631533

[4] Kessler D. The sixth stage of grief. Seattle, WA: Kindle Amazon (2019).

[5] Shear MK Ghesquiere A Glickman K. Bereavement and complicated grief. Curr Psychiatry Rep. (2013) 15:1–7. doi: 10.1007/s11920-013-0406-z PMC3855369 24068457

[6] Cuijpers P Geraedts AS van Oppen P Andersson G Markowitz JC van Straten A. Interpersonal psychotherapy for depression: a meta-analysis. Am J Psychiatry. (2011) 168:581–92. doi: 10.1176/appi.ajp.2010.10101411, PMID: 21362740 PMC3646065

[7] Bleiberg KL Markowitz JC. Interpersonal psychotherapy for PTSD: treating trauma without exposure. J Psychother Integr. (2019) 29:15–22. doi: 10.1037/int0000113, PMID: 31534308 PMC6750225

[8] Lotzin A Acquarini E Ajdukovic D Ardino V Böttche M Bondjers K. Stressors, coping and symptoms of adjustment disorder in the course of the COVID-19 pandemic – study protocol of the European Society for Traumatic Stress Studies (ESTSS) pan-European study. Eur J Psychotraumatol. (2020) 11:1780832. doi: 10.1080/20008198.2020.1780832, PMID: 33029321 PMC7473046

[9] Partis M. Bowlby’s attachment theory: implications for health visiting. Br J Community Nurs. (2000) 5:499–503. doi: 10.12968/bjcn.2000.5.10.12988, PMID: 12181518

[10] Grey N Holmes EA. Hotspots in trauma memories in the treatment of post-traumatic stress disorder: a replication. Memory. (2008) 16:788–96. 18720224 10.1080/09658210802266446

[11] Brewin CR Andrews B Valentine JD. Meta-analysis of risk factors for posttraumatic stress disorder in trauma-exposed adults. J Consult Clin Psychol. (2000) 68:748–66. doi: 10.1037//0022-006x.68.5.748, PMID: 11068961

[12] Brewin CR Dalgleish T Joseph S. A dual representation theory of posttraumatic stress disorder. Psychol Rev. (1996) 103:670–86. doi: 10.1037/0033-295x.103.4.670, PMID: 8888651

[13] Pini S Gesi C Abelli M Muti M Lari L Cardini A. The relationship between adult separation anxiety disorder and complicated grief in a cohort of 454 outpatients with mood and anxiety disorders. J Affect Disord. (2012) 143:64–8. doi: 10.1016/j.jad.2012.05.026, PMID: 22832169

[14] Smid GE Kleber RJ de la Rie SM Bos JBA Gersons BPR Boelen PA. Brief eclectic psychotherapy for traumatic grief (BEP-TG): toward integrated treatment of symptoms related to traumatic loss. Eur J Psychotraumatol. (2015) 6:27324. doi: 10.3402/ejpt.v6.27324, PMID: 26154434 PMC4495623

[15] Shear K Frank E Houck PR Reynolds CF. Treatment of complicated grief: a randomized controlled trial. JAMA. (2005) 293:2601–8. doi: 10.1001/jama.293.21.2601 15928281 PMC5953417

[16] Miller MD Frank E Cornes C Imber SD Anderson B Ehrenpreis L. Applying interpersonal psychotherapy to bereavement-related depression following the loss of a spouse in late life. J Psychother Pract Res. (1994) 3:149–62. PMID: 22700188 PMC3330363

[17] Nelson JC Pikalov A Berman RM. Augmentation treatment in major depressive disorder: focus on aripiprazole. Neuropsychiatr Dis Treat. (2008) 4:937–48. doi: 10.2147/ndt.s3369 19183784 PMC2626914

